# Rhein Protects Against Neurological Deficits After Traumatic Brain Injury in Mice *via* Inhibiting Neuronal Pyroptosis

**DOI:** 10.3389/fphar.2020.564367

**Published:** 2020-09-30

**Authors:** Fangfang Bi, Huaifen Ma, Chen Ji, Cuicui Chang, Wenbo Liu, Keliang Xie

**Affiliations:** ^1^ Department of Medicine, Xi’an Peihua University, Xi’an, China; ^2^ College of Anesthesiology, Weifang Medical University, Weifang, China

**Keywords:** Rhein, traumatic brain injury, inflammatory cytokines, pyroptosis, neurological deficits

## Abstract

Neurological dysfunction provoked by traumatic brain injury (TBI) makes a huge impact on individual learning ability, memory level, social participation, and quality of life. Pyroptosis, the caspase-1-dependent cell death, which is associated with the release of numerous pro-inflammatory factors, plays a major role in the pathological process after TBI. Inhibition of pyroptosis has been shown to be an attractive strategy for the treatment of various neurological disorders. Here, we found that Rhein, an anthraquinone derived from the medicinal plant rhubarb, attenuated TBI-induced upregulation of pro-inflammatory cytokines, blood lactate dehydrogenase (LDH), and pyroptosis-related proteins, as well as reduced neurological dysfunction in a mouse TBI model. Consistently, Rhein inhibitd equiaxial stretch-induced neuron pyroptosis, LDH release, and upregulation of pro-inflammatory factors *in vitro*. Thus, our study suggested that Rhein protected against neurological deficits after TBI *via* inhibiting neuronal pyroptosis.

## Introduction

Traumatic brain injury (TBI), occurs when the brain is exposed to external forces, has become the main cause of death and disability in young adults with an increasingly year-prevalence. As the epidemiological investigation suggested, TBI will be a critical global health issue and a major cause of disabilities by 2020 (Peeters et al., 2015; Brazinova et al., 2018). Neurological dysfunction caused by TBI shows great impact on individual learning ability, memory level, social participation, and quality of life (Chen et al., 2018). Despite the considerable researches on TBI, there still lacks of effective therapeutic treatments in the clinic. Therefore, further delineating the pathogenic mechanism underlying TBI is essential for the development of new therapeutic strategies.

Inflammatory response was reported to plays an important role in the pathological process after TBI, including neuronal death, oxygen-free radical formation, calcium release, and mitochondrial dysfunction (Angeloni et al., 2015; Hiebert et al., 2015; Corrigan et al., 2016; Russo and McGavern, 2016; Esterov and Greenwald, 2017). The chemical neurotoxicity can also induce cortical neuron reduction (Li et al., 2017). Besides, previous researches indicated that neuronal cells would lost after TBI (Liu et al., 2018). Proinflammatory factors can cause pyroptosis, which is characterized by the swelling, dissolution of cells and the release of proinflammatory cytokines and intracellular contents. This specific type of necrosis is involved in the myocardial ischemia, lung and kidney damage, and stroke (Zhao et al., 2011; Meng et al., 2013; Zeng et al., 2013). Recent studies found pyroptosis was not only in macrophages but also in dendritic cells and other types of cells. Cellular pyroptosis is mediated by two cysteine-containing aspartate proteolytic enzymes (caspase), including caspase-1, 4, 5, 11 (4, 5 presents in humans). These proteolytic enzymes belong to inflammatory protein, playing a key role in the immune response and serving as an important component of the “inflammasome”. Activation of these caspases induces cell pyroptosis and inflammasome activation, which has an important role in endotoxic shock and Gram-negative bacterial-induced sepsis (Shi et al., 2014). Evidence shows that gene *gasderminD* is a key substrate for caspases (Shi J. et al., 2015). The amino terminal peptide of caspases cleaved by gasdermin D can provoke cell death and secretion of inflammatory factors, revealing the key molecular of pyroptosis.

Rhein (1.8-dihydroxy-3-carboxy-anthraquinone) is an anti-inflammatory active ingredient enriched in rhubarb. It is widely used to treat various inflammatory diseases in the clinical (Zeng et al., 2014). As a precursor of Rhein, the oral drug Diacerein shows it therapeutic effects on disease like by metabolizing into Rhein. Through blocking TLR-related signaling pathways, Diacerein commits the anti-inflammatory function of Rhein (Yu et al., 2015; Zhang et al., 2015). Compared with most non-steroidal anti-inflammatory drugs, Diacerein possesses gastrointestinal protection for it does not affect the production of prostaglandin E2 (PGE2) (Fernand et al., 2011; Dhaneshwar et al., 2013).

In the present study, we applied an *in vivo* mouse model of TBI and *in vitro* cellular models of mechanical stress to investigate the protection of Rhein on TBI neurological function and its inhibitory effect on TBI-induced neuronal burnout. We indicated that Rhein can relieve neurological deficits in TBI mice by reducing the death of neurons. It is a promising finding that may further our current understanding of the brain-protective role of Rhein. Together, our study showed a new protective effect of Rhein and revealed the potential of Rhein in the clinical treatment of TBI.

## Materials and Methods

### Establishment of the TBI Models

Animal experiments were conducted in accordance with the National Institutes of Health guide for the care and use of laboratory animals (NIH Publications No. 8023, revised 1978). This study was approved by Ethics Committee of Medical College of Xi’an Peihua University. Male C57BL/6 mice aged 8 weeks were purchased from the model animal research center of Xijing Hospital. These mice were housed in the specific pathogen-free (SPF) conditions with the standard temperature (22 ± 1°C), humidity (50–60%), and light conditions (12 h light/dark cycle). Mouse model of TBI was adopted from a previous study (Liu et al., 2018). Briefly, Mice were anesthetized with the intraperitoneal injection of 5% chloral hydrate (0.08 ml/10g), then fixed in a stereotaxic device, shaved and cleaned the scalp with iodophor, and exposed the left lateral aspect of skull. TBI models were established by fall a 200-g steel weight with a flat end from a height of 5 cm into the left lateral skull.

According to a previous reports (Shi H. et al., 2015; Dong et al., 2018), 15 min after TBI, mice received an intraperitoneal injection of Rhein. And the dose of Rhein (100 mg/kg) was determined in the preliminary experiment (**Supplementary Figure**). Mice were basically assigned to 4 groups: (1) Sham: PBS injection, n = 8 in group; (2) Rhein: mice receiving Rhein, n = 12 in group; (3) TBI: n = 12 in group; and (4) Rhein/TBI: mice were injected with Rhein 15 min after TBI, n = 12 in group. After experiment completion, mice were euthanized by CO_2_ inhalation (the flow rate of CO_2_ displaced 20% of the chamber volume per minute) and mouse brains were stored at -80°C for further analysis.

### Neurobehavioral Training and Evaluation

Modified neurological severity scoring (mNSS), open-field, and Rotarod testing were used to assess neurological deficits 8, 24, and 48 h after TBI (Liu et al., 2018). The mNSS trial composed of ten different tasks that can evaluate the motor (muscle status and abnormal movement), sensory (visual, tactile, and proprioceptive), balance, and reflex functions of mice. Neurological deficits was graded from 0 to 18 (0 = normal; 18 = maximal deficit). One point was scored for each abnormal behavior or for the lack of a tested reflex. The open-field trial based on the pattern of exploration (center vs. periphery) was used to assess anxiety-like behavior. Mice were tracked under moderate lighting for 15 min in a 40-cm^2^ open field using software (ANY-Maze, Stoelting, USA). General activity was assessed by fixing the total of distance traveled. Rotarod trial were used to assess motor coordination and learning. On testing day, the mice were given four 300-s accelerating Rotarod tests with an intertrial interval of 30 min. The average latency to the first fall off the rod was recorded. All experimenters were blinded to four group mice.

### Primary Neuronal Culture and Injury Models

Mice (embryo 13–14 days BALB/c) were decapitated, and the meninges were removed. Then the cortex was cut with ophthalmic scissors and then digested with papayotin at 37°C for 20 min. Then flocs were deal with DNase I (Sigma) after centrifugation at 1000×g for 3 min. The cells were plated on a poly-L-lysine––coated petri dish at a density of 1 × 10^6^ cells. After 4 hours, the medium was changed to neuronal basal medium, and the cultured cells on the 7th–14th day were used for the experiment. Subsequently, primary neurons were examined neuronal purity by NeuN staining.

We then examined the effects of mechanical stress on neurons *in vitro*: In the stretch model, neurons were seeded in 6-well plates (BioFLEX^®^). The equiaxial stretch (12% strain, 1.0 Hz frequency) was applied to the cultured neurons for 12 h *via* a Flexcell^®^FX-5000™ tension system (Flexcell, USA). Neurons were treated with Rhein (10 µg/ml) 1 h before stretch. Cells were used for immunofluorescence and protein or RNA extraction.

### Terminal Deoxynucleotidyl Transferase-Mediated dUTP Nick End Labeling

The cerebral cortex was collected, and apoptosis was determined by terminal deoxynucleotidyl transferase-mediated dutp nick end labeling (TUNEL) staining. TUNEL staining was performed with fluorescein-dUTP for apoptotic cell nuclei and 4′,6- diamidino-2-phenylindole (DAPI) to stain all cell nuclei by using TUNEL Apoptosis Assay Kit (R&D, Switzerland).

### Western Blotting

Proteins were drawn from the tissues and cells and quantified by using a BCA protein kit (Thermo Scientific). Proteins (50 μg) were loaded on SDS-PAGE gels per lane and transferred to PVDF membranes after electrophoresis. Then blocking membranes with 5% BSA at 2 h room temperature and incubating at 4°C overnight with the primary antibodies: GAPDH (Abcam, ab8245, 1:5,000), GSDMD (Santa Cruz Biotechnology, sc-393581, 1:1,000), caspase-11 (Abcam, ab22684, 1:1,000), caspase-1 (Abcam, ab138483, 1:1,000), caspase1 (p10), and caspase-1 (p20) (AdipoGen, AG-20B-0044, AG-20B-0042, 1:1,000). Immunoreactivity was detected by incubating with secondary antibodies (Abcam ab205718, ab97023, 1: 20,000).

### Immunohistochemistry

Fix brain tissues with 4% Paraformaldehyde solution. After fixed, each sample was dehydrated and embedded. Paraffin-embedded sections (4μm) were prepared for antigen retrieval, blocking, primary antibody incubation (GSDMD, 1: 50, Santa Cruz Biotechnology; caspase-11, 1:100, Abcam; caspase-1, 1:100, Abcam), secondary antibody incubation and staining with DAB.

### Lactate Dehydrogenase Release Detection

Mice were collected the blood serum was measured for release. The primary neurons were collected supernatant from serum-free media using 0.2-μm syringe filters. The Lactate dehydrogenase (LDH) detection was using a commercially available kit (Solarbio). Transfer serum or supernatant to a 96-well plate, then add the reaction mixture and incubate in the dark for 30 min. The LDH concentration was quantified by measuring the absorbance at 490nm.

### Quantitative Real-Time Polymerase Chain Reaction

Total RNA was drawn from the tissue or neurons using a Trizol reagent (Trizol™ Reagent, Invitrogen). Quantitative real-time polymerase chain reaction (qRT-PCR) was performed by using a SuperReal PreMix Plus Kit (SYBR Green) (Qiagen) on the Bio-Rad CFX96TM Real-Time System. GAPDH was amplified as an internal control. Primer sequences as follows (Liu et al., 2018): TLR4 F: 5’- TCACA ACTCG CCCAA GGAGG AA -3’, R: 5’- AAGAG ACCAC GGCAG AAGCT AG -3’; MyD88 F: 5’- CCACC TGTAA AGGCT TCTCG -3’, R: 5’-CTAGA GCTGC TGGCC TTGTT-3’; NLRP3 F: 5’- GCTAA GAAGG ACCAG CCAGA GT -3’, R: 5’- GAACC TGCTT CTCAC ATGTC GT -3’; GAPDH F: 5’- AACTT TGGCAT TGTGG AAGG -3’ R: 5’- GGATG CAGGG ATGAT GTTCT -3’.

### Enzyme-Linked Immunosorbent Assay

The concentrations of total protein were measured using the BCA Protein Assay Kit (Thermo Fisher Scientific). The levels of IFN-γ, IL-1β, and IL-18 were measured using ELISA kits (Anoric-Bio) according to the manufacturer’s instructions.

### Statistical Analysis

All data are represented as means ± SEM and analyzed by SPSS statistical software. mNSS test were analyzed using the Kruskal-Wallis H analysis followed by a Mann-Whitney U test. Rotarod data were analyzed using the One-way analysis of variance (ANOVA) with repeated measures. The remaining biochemical data were analyzed using a two-way ANOVA. Each experiment was repeated three times, and Statistical differences were analyzed using the two-tailed Student’s t test or one-way ANOVA. P < 0.05 was statistically significant.

## Results

### Rhein Attenuated TBI-Induced Neurological Functional Impairment

**Figure 1:**
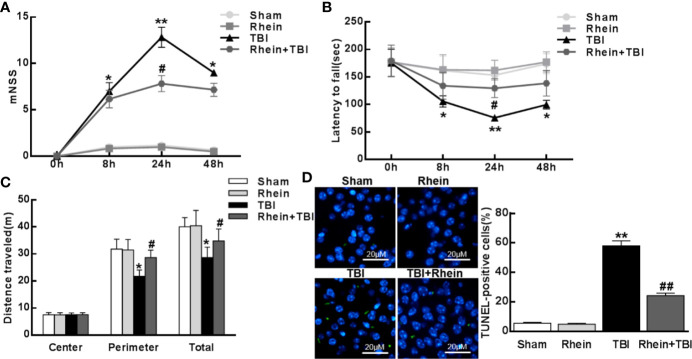
Rhein attenuated TBI-induced neurological functional impairment. The Neurological impact of TBI was tested by mNSS **(A)**, rotarod **(B)**, and open-field **(C)** behavioral task tests before and 8, 24, and 48 h after TBI. **(D)** TUNEL staining was applied to detect cortical harm in TBI. TUNEL-positive cells (%), the number of TUNEL-positive cells divided by the total cells per field, was assessed in 20 randomly selected fields. Data are presented as the mean ± SEM. *p < 0.05, ** p < 0.01 versus Sham group, ^#^p < 0.05, ^##^p < 0.01 versus TBI group.

To examine neurological functional impairment, we assessed mNSS, Rotarod, and open-field behavioral task tests before and 8, 24, and 48 h after TBI. There was no significant difference between Sham group and Rhein group in mNSS and Rotarod score (**Figures 1A, B**). However, mNSS scores peaked at 24 h and mildly decreased at 48 h. Rhein + TBI group displayed lower mNSS scores compared with TBI group (**Figure 1A**). The residence time of mice on the rotarod bottomed out at 24 h and slightly increased at 48 h in TBI group. Compared with TBI group, the residence time on the rotarod in Rhein + TBI group prolonged with the increasing motor latency (**Figure 1B**). The open-field behavioral experiment showed that the Sham group and Rhein group mice spent more time in the perimeter zone and had longer total travel distance. A significant difference between TBI and Rhein + TBI group in the perimeter zone and total travel distance was observed at 24 h after TBI (**Figure 1C**). TUNEL staining was applied to detect cortical damage in mice. No significant difference was observed between the Sham group and Rhein group. Apoptosis index, the number of TUNEL-positive cells divided by the total cells, was remarkably enhanced in TBI group but decreased in Rhein + TBI group 24 h after TBI (**Figure 1D**). In conclusion, Rhein eased TBI-induced neurological deficits.

### Rhein Reduced Levels of Inflammatory Mediator in the Cortex After TBI

**Figure 2:**
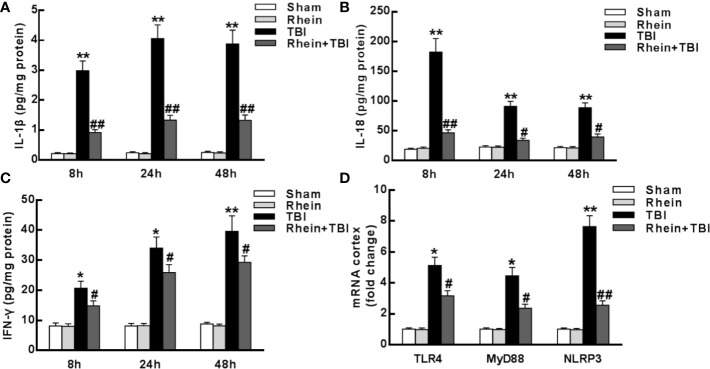
Rhein reduced levels of inflammatory mediator in the cortex after TBI. **(A–C)** Concentrations of pro-inflammatory cytokines IL-1β **(A)**, IL-18 **(B)**, and IFN-γ **(C)** were detected in the region of the contusion 8, 24, and 48 h after TBI by ELISA. **(D)** TLR4, MyD88, and NLRP3 mRNA expression were tested by qRT-PCR. GAPDH served as an internal control. Data are presented as the mean ± SEM. *p < 0.05 and **p < 0.01 versus Sham group, ^#^p < 0.05 and ^##^p < 0.01 versus TBI group.

The changes of the inflammatory-related mediators in brain tissue was tested with ELISA. Lower levels of the pro-inflammatory cytokines IL-1β, IL-18, and IFN-γ were observed in Sham group and Rhein group compared with other groups. All pro-inflammatory cytokines were augmented in TBI group but inhibited in Rhein + TBI group (**Figures 2A–C**). Consistent with the protein levels, the mRNA expression of TLR4, MyD88, and NLRP3 in the injured brains 24 h after TBI was lower in Sham group and Rhein group than in other groups. The levels of these factors were increased in TBI group but significantly reduced in Rhein + TBI group (**Figure 2D**). Together, these results suggested that Rhein inhibited TBI-induced pro-inflammatory mediator production in the cortex.

### Rhein Attenuated Pyroptosis in the Murine Model of TBI

**Figure 3:**
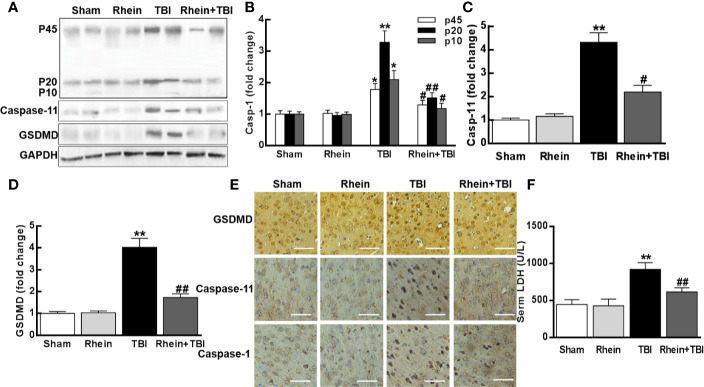
Rhein attenuated pyroptosis in the murine model of TBI. **(A–D)** Expression of pyroptosis-related proteins of caspase-1 (p45, p20, and p10), caspase-11, and GSDMD was assayed by Western blot 24 h after TBI (two randomly selected samples from each group were shown). **(E)** Representative photomicrographs at × 20 magnification of proteins immunostaining in the cortex 24 h after TBI. **(F)** Serum LDH was tested 24 h after TBI. Data are presented as the mean ± SEM. *p < 0.05 and **p < 0.01 versus Sham group, ^#^p < 0.05, ^##^p < 0.01 versus TBI group.

The expression of pyroptosis-related proteins was assessed by Western Blot (**Figures 3A–D**). Compared to Sham and Rhein group, caspase-1 (including p10, p20, and p45), caspase-11, and GSDMD were severally increased 24 h after TBI. Rhein + TBI group reduced the enhancement of TBI-induced proteins. Immunostaining showed that caspase-1, caspase-11, and GSDMD were highly expressed at 24 h in TBI group mice, while they were less expressed in Rhein + TBI group mice (**Figure 3E**). As cerebral injury often provokes cellular LDH leakage, we tested serum LDH after TBI. In contrast to Sham group and Rhein group, Serum LDH concentration was increased in TBI group while was nearly normal levels in Rhein + TBI group (**Figure 3F**). These results suggested that Rhein effectively inhibited pyroptosis in the murine model of TBI.

### Rhein Reduced Neuron Injury-Induced Inflammatory Mediator Levels *In Vitro*

**Figure 4:**
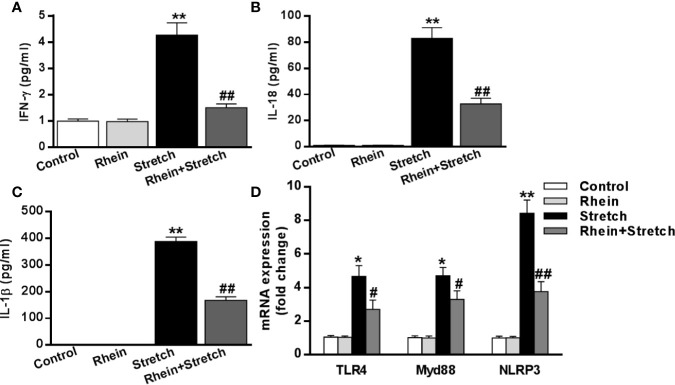
Rhein reduced neuron injury-induced inflammatory mediator levels *in vitro*. The neurons were treated with stretch stimulation for 12 h in absence or presence of Rhein (10 µg/ml), then the expression of pro-inflammatory cytokines IFN-γ **(A)**, IL-18 **(B)**, and IL-1β **(C)** were tested by ELISA. mRNA expressions of TLR4, MyD88, and NLRP3 after stretch and Rhein treatments were detected by qRT-PCR **(D)**. Data were presented as three independent experiments. *p < 0.05, **p < 0.01 versus control group; ^#^p < 0.05, ^##^p < 0.01 versus stretch group.

The expression of inflammatory mediators after stretch stimulation was detected by ELISA. Compared to Control group and Rhein group neurons, pro-inflammatory cytokines (IFN-γ, IL-1β, and IL-18) were enhanced significantly in stretch group neurons while were inhibited in Rhein + stretch group neurons (**Figures 4A–C**). The mRNA expression was assessed using qRT-PCR. In contrast to Control group and Rhein group, levels of these transcripts (*TLR4*, *MyD88*, and *NLRP3*) were increased markedly in stretch group neurons but were reduced in Rhein+Stretch group neurons (**Figure 4D**). These results indicated that Rhein inhibited neuron injury-induced inflammatory mediator levels *in vitro*.

### Rhein Ameliorated Neuron Injury-Induced Pyroptosis

**Figure 5:**
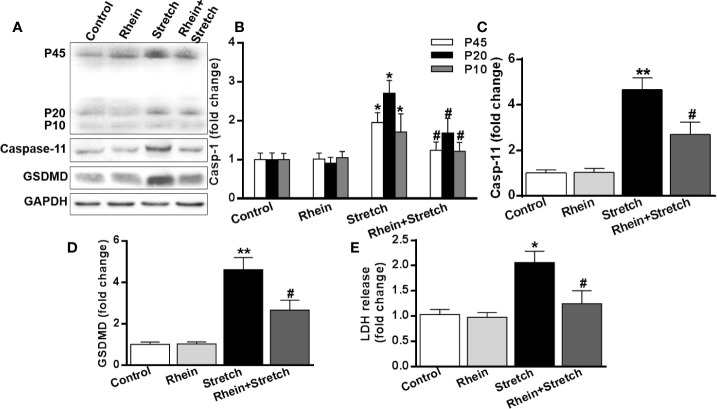
Rhein ameliorated neuron injury-induced pyroptosis. The neurons were treated with stretch stimulation for 12 h in the absence or presence of Rhein (10 µg/ml), then cell lysate and culture supernatant were collected. **(A–D)** Pyroptosis-related protein expression was detected by Western blotting. The histogram was used to analyze protein expression. **(E)** Supernatant LDH concentration was detective after stretch and Rhein treatments. The statistics were based on at least three independent experiments. *p < 0.05, **p < 0.01 versus control group; ^#^p < 0.05 versus Stretch group.

To uncover the correlation between neuron injury and pyroptosis, the expression of pyroptosis-related proteins was detected with Western Blot. The expression of caspase-1, caspase-11, and GSDMD of neurons were enhanced in Stretch group but were decreased in Rhein + stretch group (**Figures 5A–D**). Similar results were also obtained in supernatant LDH concentration assay (**Figure 5E**), indicating that Rhein ameliorated neuron injury-induced pyroptosis.

## Discussion

In this study, we evaluated the damage on sensory dysfunction and motor dysfunction after TBI. The results showed a higher mNSS score and the lower Rotarod test score at 24 h. We found that TBI mice significantly enhanced neuronal dysfunction at 24 h. Previous study observed that neurological deficit of TBI mice was worse at 24 h than at 72 h (Xu et al., 2017). Then we detected inflammatory correlation factors in the acute phase. A series of pro-inflammatory cytokines (IL-1β, IFN-γ, and IL-18) secretion were found to increase after TBI. Besides, mRNA expression of the upstream inflammatory regulatory molecules including TLR4, MyD88, and NLRP3 increased at 24 h. The protein levels of caspase-1, caspase-11, and GSDMD in damaged cortical tissue and blood LDH release also enhanced 24 h after TBI. As mechanical stretch was used to study bone-related and cardiovascular diseases (Yu et al., 2016), we established a neuronal injury model under equiaxed stretch at the cellular level (1.0 Hz frequency, 12% strain, and 12 h), The results of *in vitro* experiments supported the findings of *in vivo* experiments. These data suggested that pyroptosis was likely involved in neuroinflammation with TBI.

We previously found that inflammation in the mouse cerebral cortex gradually increased with the progress of TBI. Significant neurological damage and upregulation of caspase-1, caspase-11 and GSDMD were also observed in our study. Caspase-1 knockout TBI mice showed remarkably reduced neuroinflammation, neuronal damage, and neurological dysfunction compared with the normal group, indicating that neuronal pyroptosis was an important mechanism of neuronal death following injury (Liu et al., 2018). Cellular pyroptosis is mediated by two cysteine-containing aspartate proteolytic enzymes (caspase), including caspase-1, 4, 5, 11 (4, 5 presents in humans). These proteolytic enzymes belong to inflammatory protein, playing a key role in immune response and serving as an important components of the “inflammasome”. Activation of these caspases induces cell pyroptosis and inflammasome activation, which has an important role in endotoxic shock and Gram-negative bacterial-induced sepsis (Shi et al., 2014). In addition, these caspases show impact on the development of infectious diseases, nervous system-related diseases, and atherosclerotic diseases. Studies found that gene *gasderminD* is a key substrate for these caspase in 2015 (Shi J. et al., 2015). The amino terminal peptide of these caspase cleaved by gasdermin D could cause cell death and provoke secretion of inflammatory factors, which may be the key molecular mechanism of pyroptosis. Recently, Meng et al. showed that inflammasome NLRP1 is highly expressed in neurons and is associated with a variety of neurological diseases. Activated NLRP1 causes inflammatory responses and cell pyroptosis (Meng et al., 2014). Targeted inhibition of NLRP1 attenuates intrinsic immune response, neuronal death, and age-related cognitive impairment in various animal models (Mawhinney et al., 2011). Neuroinflammation occurs throughout the range of central nervous system lesions (Helmy et al., 2011). It has been recognized that drug therapy can modulate inflammation to control TBI damage (Thelin et al., 2017). Moreover, The antagonists of NLRP3 inflammasome and IL-1 receptor reduce neuroinflammation following TBI (Irrera et al., 2017). Blocking electroacupuncture-induced TLR4 signaling promotes hippocampal neurogenesis and nerve recovery post-traumatic (Ye et al., 2017).

Rhein is an anti-inflammatory active ingredient enriched in rhubarb. It has been found that Rhein inhibited IL-1β-induced activation of NF-κB and AP-1 in hypoxic cultured chondrocytes (Martin et al., 2003). In human umbilical vein endothelial cells, Rhein restrained the production of vascular cell adhesion factor-1 (Hu et al., 2013). It also prevented endotoxin-induced acute kidney injury through suppressing the activity of NF-κB (Yu et al., 2015) and alleviated acute kidney injury caused by sepsis *via* blocking the TLR4 pathway (Zhang et al., 2015). In addition, Rhein improved chronic kidney disease in rats (Su et al., 2013), showed anti-tumor effect (Tsang and Bian, 2015), displayed anti-imbalance oxidative stress and anti- fibrosis function (Guo et al., 2003). In KK/HIJ diabetic rats (non-alcoholic fatty liver disease), Rhein reduced inflammation and fat infiltration (Wei et al., 2016), along with curing intervertebral disc degeneration (Li et al., 2011). Moreover, Rhein protected renal by upregulating the expression of Klotho in kidney fibrosis model mice (Zhang et al., 2016; Zhang et al., 2017). In the present study, we treated mice with intraperitoneal injection of Rhein. Rhein was found to inhibit the production of inflammatory factors, the mRNA expression of TLR4, MyD88, and NLRP3, and the protein levels of caspase-1, caspase-11, and GSDMD. Rhein also alleviated neurological deficits in mice after TBI. Our research demonstrated that Rhein alleviates neurological deficits by improving TBI-induced neuronal burnout. This finding may provide a strategy for therapeutic treatment of TBI in clinical.

In conclusion, our study found that Rhein relieved neurological deficits by suppressing TBI-induced neuronal pyroptosis. Rhein exerted an anti-inflammatory effect in damaged cortex, which inhibited TBI-induced neuronal pyroptosis and neurological deficits.

## Data Availability Statement

The raw data supporting the conclusions of this article will be made available by the authors, without undue reservation.

## Ethics Statement

The animal study was reviewed and approved by Ethics Committee of Medical College of Xi’an Peihua University.

## Author Contributions

FB and CJ carried out the experimental work and the data collection and interpretation. CC and HM participated in the design and coordination of experimental work, and acquisition of data. KX and WL carried out the study design, the analysis, and interpretation of data and drafted the manuscript. All authors contributed to the article and approved the submitted version.

## Funding

This study was supported by a grant from the Natural Science Basic Research Program of Shaanxi (program no. 2018JM7140).

## Conflict of Interest

The authors declare that the research was conducted in the absence of any commercial or financial relationships that could be construed as a potential conflict of interest.

## Abbreviations

TBI, traumatic brain injury; TLR4, Toll-like receptor 4; MyD88, myeloid differentiation factor 88; IFN-γ, Interferon-gamma; IL-1β, Interleukin-1β; IL-18, Interleukin-18.
