# Supplementary figures and images for: Rhein Protects Against Neurological Deficits After Traumatic Brain Injury in Mice via Inhibiting Neuronal Pyroptosis
Source: Front Pharmacol. 2020 Sep 30;11:564367. doi: 10.3389/fphar.2020.564367 (PMC7554525; doi:10.3389/fphar.2020.564367)

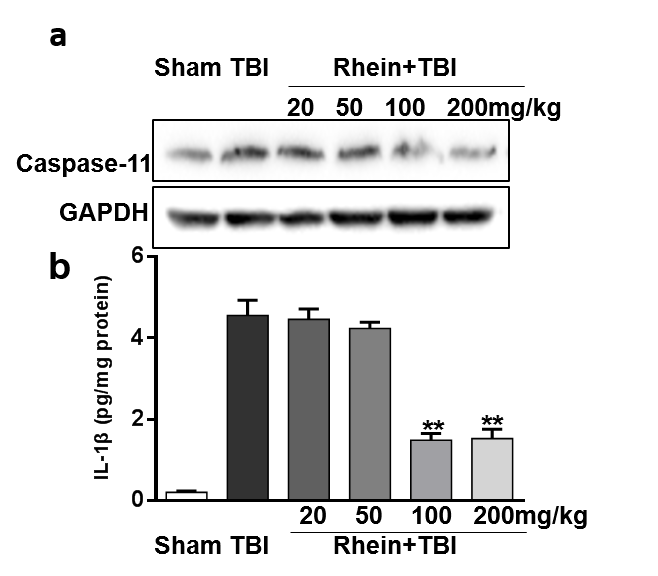

Supplement: Supplementary file 1 [file Image_1.tif]
